# Molecular Behavior of Human β Defensin Type 3 Embedded in Different Model Lipid Membranes

**DOI:** 10.1021/acs.jcim.5c02937

**Published:** 2026-04-30

**Authors:** Jackson Penfield, Tongye Shen, George R. Rucker, Liqun Zhang

**Affiliations:** † Department of Chemical Engineering, Tennessee Technological University, Cookeville, Tennessee 38505, United States; ‡ Department of Biochemistry and Cellular and Molecular Biology, University of Tennessee, Knoxville, Tennessee 37996, United States; § Chemical Engineering department, 4260 University of Rhode Island, Kingston, Rhode Island 02881, United States; ∥ Department of Chemical Engineering, Hampton University, Hampton, Virginia 23669, United States

## Abstract

Human β defensin type 3 (hBD-3) is recognized as one of the most intriguing antimicrobial peptides (AMPs) that holds the promise of solving drug resistance issues. hBD-3 can function (disruption of membrane integrity) in high salt environments, where most other AMPs fail. However, its functional mechanism at the molecular level remains elusive. To characterize its structure and dynamics during membrane crossing, long-time (a total of 57.0 μs) all-atom molecular dynamics simulations were conducted on hBD-3 monomers and dimers in both wild-type and analog (in which all three disulfide bonds are broken) forms that are embedded in four types of lipid membranes. Trajectory analysis was carried out using a statistical method—conformational dynamics analysis to calculate contact matrices and then principal component analysis (PCA) and linear discriminant analysis (LDA), in order to discern structural changes upon various physical and chemical perturbations. The result shows that the major collective coordinate primarily distinguishes between the wild-type and analog forms of hBD-3. For the hBD-3 monomer, the analog undergoes significant structural loss due to the lack of stabilizing disulfide bonds; salt exerts a nearly consistent effect on the contact degrees of freedom of the protein, whereas changes in lipid membrane composition have an insignificant effect. For the hBD-3 dimer, no consistent relationship between structure and salt concentration is indicated, and variations in the chemical composition of model bacterial membranes have a limited effect on its dynamics. These results suggest that the wild-type and analog forms of hBD-3 may employ different mechanisms when crossing bacterial membranes. The effect of salt on hBD-3 dynamics can be mitigated by the high net charge density of the protein. Additionally, the hBD-3 dimer can distinguish between model Gram-positive and Gram-negative membranes, whereas the monomer cannot. Overall, these findings provide unique insights into the structure, dynamics, and membrane-disrupting mechanism of hBD-3.

## Graphical abstract

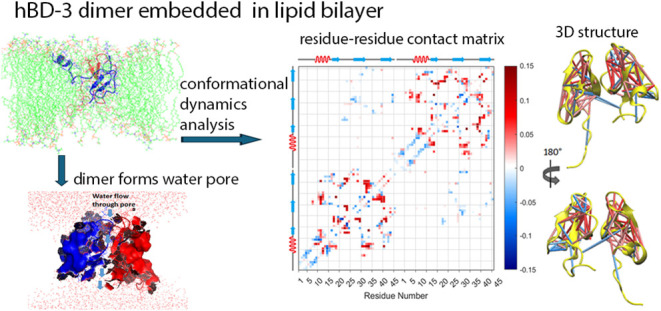

## 1 Introduction

As natural antibiotics, human defensins are secreted by the human innate immune system.^1–3^ They are cationic, cysteine-rich small peptides with lengths ranging from 21 to 46 amino acid residues.^4^ Human defensins exhibit bactericidal activity against a wide range of microorganisms, including Gram-positive (G+) and Gram-negative (G-) bacteria, yeast, fungi, some encapsulated viruses, and even SARS-CoV-2.^5–10^ Defensins achieve their antimicrobial effects by first binding to the bacterial lipid membrane, creating pores or otherwise disrupting the cell membrane of target organisms, which leads to the release of cellular contents^11,12^ and ultimately bacterial death. Because the bacterial cell membrane has a relatively stable chemical composition, the antibacterial activity of human defensins does not cause drug resistance. Therefore, they hold promise as valuable therapeutic options for the continued development of improved antibiotic biotechnology to solve infectious diseases and antibiotic resistance issues.^13^

Based on their specific disulfide bonding patterns and sizes, human defensins can be classified into α and β categories. Both human α defensins (HDs) and human β defensins (hBDs) contain six cysteine residues that form three pairs of disulfide bonds. Human α defensins form disulfide bonds in the patterns of Cys(1)-Cys(6), Cys(2)-Cys(4), and Cys(3)-Cys(5), whereas human β defensins form disulfide bonds in the patterns of Cys(1)-Cys(5), Cys(2)-Cys(4), and Cys(3)-Cys(6). These disulfide bonds can restrain the defensin structure but can break sequentially under reducing conditions, converting hBD-3 from its wild-type form into a linear analog.^14^ Among the identified human β defensins (hBDs), hBD type 3 (hBD-3) is mainly secreted by epithelial cells.^15,16^ It consists of 45 residues and carries a net charge of +11e. hBD-3 exhibits broad-spectrum antibacterial activity, in addition to chemotactic activity,^17,18^ and has also been implicated in cancer progression.^19–22^ It is generally believed that hBD-3 kills bacteria by disrupting the bacterial lipid membrane, forming aggregates within the membrane, and causing cell leakage; however, its functional mechanism at the molecular level remains unclear.

hBDs exhibit different potencies against G+ and G- bacteria,^23^ and the activity of some hBDs is inhibited at high salt conditions.^24–26^ In contrast, hBD-3 is unique and functionally distinct in that it remains active against both G+ and G- bacteria even at high salt concentrations.^27^ Moreover, hBD-3 is more potent than hBD-1 and hBD-2 in disrupting bacterial membranes,^28,29^ and it is active in both wild-type and linear analog forms.^30^ hBD-3 can form dimers in solution at lower concentrations than other hBDs, such as hBD-1 and hBD-2.^29^ Two hBD-3 molecules form a dimer through hydrogen bonding between Q29 residues.^29^ Based on μs-long all-atom molecular dynamics (MD) simulations of the hBD-3 dimer in solution, the dimer structure was found to be stable.^14^ However, breaking the disulfide bonds affects the dimer interface, rendering the dimer structure less stable. Compared with the wild-type form, the hBD-3 analog is more hydrophobic.^14^ Studying the translocation free energy of hBD-3 in monomer, dimer, and tetramer forms crossing model bacterial membranes using umbrella-sampling simulation methods, results suggest that hBD-3 may cross the membrane in an oligomeric form.^31,32^ hBD-3 monomers must overcome a higher energy barrier to cross the membrane than dimers.^31^ To gain insight into its functional activity, it is therefore important to study the dynamic characteristics of hBD-3 when crossing different bacterial membranes.

To investigate the dynamic motions and possible allosteric communication arising from residue–residue contacts within hBD-3 monomers and dimers as well as interactions between hBD-3 and surrounding lipid membranes, we conducted a total of 57.0 μs of all-atom MD simulations of hBD-3 embedded in different model membranes. In addition to analyzing water pore formation within the hBD-3 dimer, water translocation across the membrane, and aggregation of negatively charged lipids around hBD-3, we calculated covariance matrices of residue–residue contact interactions, followed by principal component analysis (PCA) and related statistical transformations based on the simulation trajectories. Statistical analysis from the perspective of contact matrices can reveal essential protein conformational dynamics that are directly connected to contact energetics. Contact analysis was performed using the program CAMERRA (Computation of Allosteric Mechanism by Evaluating Residue–Residue Associations),^33^ which extracts essential collective motions by analyzing the covariance matrix of contact coordinates. The program reads simulation snapshots, tracks the dynamic correlations of residue–residue contacts, and identifies intricate essential motions at the level of contact formation and breaking.^34^ Contact analysis has previously been applied to small to moderate-sized systems to predict allosteric communication in the context of ligand association.^35–37^ These types of statistical analyses have proven to be useful for capturing signaling motions induced by perturbation and conformational switches. In this work, we extend this approach to discern protein conformations within various lipid membrane environments. Data interpretation was first aided by contact PCA and further refined using contact linear discriminant analysis (LDA) methods. LDA is a widely used statistical method for identifying linear combinations of features that best discern two or more classes of labeled objects or events. Here, this supervised learning method was employed to identify function-related motions.

## 2 Methods

### 2.1 Simulation Details

The structure of hBD-3 monomer is available with PDB ID1KJ6.^29^ The initial dimer structure of hBD-3 was predicted in previous work,^14^ which agrees with the solution NMR-derived dimer structure reported by Schibli et al.^29^ In the linear analog form of hBD-3, all three disulfide bonds are disconnected compared with the wild-type structure. To investigate the effect of membrane chemical composition on the structure and dynamics of proteins embedded in lipid bilayers, four types of negatively charged lipid bilayers were constructed in this study: (i) POPE and POPG mixed bilayer with a POPE:POPG ratio of 3:1 to represent G- bacterial membrane; (ii) POPE and POPG mixed bilayer with a POPE:POPG ratio of 1:3 to represent G+ bacterial membrane; (iii) POPC bilayers containing 10% phosphatidylinositol 4,5-bisphosphate (PIP2); and (iv) POPC and POPG mixed bilayer with a POPC:POPG ratio of 3:1. Each system contained a total of 200 lipids, with 100 lipids in the upper leaflet and 100 lipids in the lower leaflet. The initial systems of hBD-3 monomers and dimers embedded in different lipid bilayers were constructed using CHARMM-GUI online program.^38^ The common initial structures of hBD-3 monomers and dimers within lipid bilayers are shown in Figure S1 (Left), with the main axis of the protein aligned along the membrane normal (*Z*-axis) (called Z-orientation). In addition, to increase the diversity of initial configurations, alternative structures shown in Figure S1 (Middle and Right) were also used, in which hBD-3 was aligned in a head-on orientation relative to the membrane (called the head orientation).

To examine whether the salt concentration in the solvent influences the structure and dynamics of hBD-3 within lipid membranes, systems containing both physiological salt concentration (0.15 M NaCl) and high salt concentration (0.3 M NaCl) were prepared during the solvation process. A complete list of simulations performed in this study is provided in Table S1 in the Supporting Information (SI).

After equilibration, at least 100 ns of all-atom molecular dynamics simulations were performed using NAMD program^39^ with CHARMM36m force field^40^ for hBD-3 monomers in lipid bilayers. For hBD-3 dimers, at least 300 ns of NAMD simulations were conducted prior to extending the simulations on Anton,^41^ resulting in total simulation times of at least 1.5 μs for all systems (around half even reaching 2.4 μs). There are two exceptions. The first one is the dimer in analog form embedded in the POPC/POPG mixed bilayer; a total of 2.4 μs of all-atom simulation was performed on Anton2 machine following 20 ns equilibration on the local computer using NAMD program. The initial structure for this simulation is shown in Figure S1 (Left). The other system is the hBD-3 dimer in wild-type form embedded in the POPE/POPG = 1/3 bilayer in a head orientation, and a total of 4.8 μs of simulation was conducted.

To characterize the aggregation of negatively charged lipids around positively charged hBD-3 during the simulations, the number density of POPG lipids surrounding hBD-3 was calculated using CHARMM program^42^ for the first 100 ns and the last 100 ns of the 2.4 μs simulation of the hBD-3 dimer embedded in POPC/POPG bilayer. Similarly, the number densities of PIP2 or POPG lipids around hBD-3 were calculated for the first 10 ns and the last 100 ns of the μs-long simulations of the hBD-3 dimer embedded in POPC+10%PIP2 and POPE+POPG bilayers.

The Wordom program^43,44^ was applied to calculate the radius of gyration (*R*_g_) of hBD-3. The displacement of the protein center of mass (COM) relative to the lipid bilayer COM was calculated using CHARMM program.^42^ Root-mean-square deviation (RMSD) was also calculated using the CHARMM program after aligning the trajectory to the initial protein structure, and chain equivalence was not applied in the RMSD calculation for the hBD-3 dimer. The number of water molecules crossing the membrane was calculated using the CHARMM program and an in-house R program by tracking water molecule positions over time while accounting for periodic boundary conditions (PBC). For water translocation analysis, trajectory frames were saved every 2 ps, and each analysis window was covered 10 ns after equilibration. The number of hydrogen bonds formed between the protein and the lipid membrane was calculated using VMD program^45^ with a distance cutoff of 3.5 Å and an angle cutoff of 20 degrees. The pore size formed by two hBD-3 units of the dimer was calculated using the PoreWalker online program.^46^

### 2.2 Conformational Dynamics Analysis

Besides the conventional analyses of MD simulation trajectories, we examined residue–residue contact degrees of freedom (DOFs) and expressed the conformation of hBD-3 using statistical analysis of contact dynamics. An individual residue–residue contact *u_ij_* is defined as formed between residues *i* and *j* when any atom of residue i is within a distance cutoff (4.2 Å) of any atom of residue *j*. The value of *u_ij_* is assigned to 1 when the contact is formed, and 0 otherwise.^47^ One advantage of using contacts rather than metrics such as inter-residue distances to describe protein conformational dynamics is that contacts are directly connected to energetics, and this description is especially useful for characterizing large-scale biomolecular structural dynamics, ranging from protein folding and allostery to chromosome folding.^48–50^

For each conformation, the collection of all contacts forms a contact matrix *u_ij_*, which is typically expressed as a contact heatmap. Statistical analysis can be performed on such contact matrices over an ensemble of conformations. The goal of this analysis is to extract the most important degrees of freedom from the contact data. We first computed the mean contact matrix ⟨*u*⟩, where the elements ⟨*u*⟩_*ij*_ are averages over individual simulation snapshots. These elements are referred to as contact ratios or contact frequencies and are bound between 0 and 1. Largely static contacts—those that are either never formed or nearly always formed during the simulations—were removed. Specifically, we selected dynamic contacts with mean contact values between a lower bound ⟨*u*⟩_L_ and an upper bound ⟨*u*⟩_H_. A very restrictive range reduces the number of dynamic contacts and may eliminate important molecular motions, whereas an overly inclusive range can introduce noise into the analysis, as discussed previously.^51,52^

After the dynamic contacts were selected, statistical analysis was applied to identify collective contact degrees of freedom. One widely used linear method for identifying collective and dominant motions is principal component analysis (PCA). Applying PCA to contact DOFs reveals dynamic modes of the protein expressed through concerted contact formation and/or breaking.^6^ One can further generalize it from single-ensemble dynamics to the application of a mixture of ensembles. In such cases, we are not looking for the actual dynamics of specific molecules, but rather, contact PCA can still be used to find structural differences between different ensembles. Even though the collective coordinates for a combined ensemble are not true dynamic modes, they still are referred to as principal components (PCs), i.e., the normalized eigenvectors of the covariance matrix, and are ordered by decreasing importance according to their associated eigenvalues λ, yielding PC1, PC2, and so on. These PCs are also known as loadings, each providing a set of coefficients that relate the original variables to the transformed variables. When scaled by the amplitude of fluctuation, the *i*th PCA loading is given by $PC_{i}\times \sqrt{\lambda_{i}}$ . PCA yields a linear transformation of the contact DOFs based solely on covariance analysis. Individual conformations can be represented by projecting them onto the PCs, producing transformed coordinates known as PC scores. These dominant PCs capture modes with the largest amplitudes of fluctuation, and they do not always have a direct one-to-one correspondence to specific states of biological interest such as differences between wild-type and reduced analog states, variations in salt concentration, or interactions with different lipid bilayers. Therefore, we further employed a supervised machine learning algorithm, linear discriminant analysis (LDA),^53^ to discern the conformations under low and high salt conditions, between wild-type and analog forms, and across different lipid membrane environments. In this case, each LDA mode (LD1, LD2, etc.) represents a further linear transformation of the top PC projections. The goal of this series of transformations of coordinates (Cartesian to contact to PCA and LDA) is to project different structural ensembles onto one common latent space.

To generate residue-pair contact data, μs-long trajectories from 28 simulations (most of which were 2.4 μs in length) were exported at a rate of 2000 frames per 2.4 μs of simulation time, corresponding to a temporal resolution of 1.2 ns per frame. This resulted in a total of at least 18,775 frames for the monomer and 21,390 frames for the dimer. Each frame contained only the hBD-3 monomer or dimer, consisting of 45 or 90 residues and 744 (monomer) or 1476 (dimer) atoms, respectively. Because this study focuses on dynamic changes in protein structure, residue-pair contact DOFs that were formed in more than 90% or less than 10% of the frames were considered static and excluded from further analysis. Accordingly, the upper and lower bounds for the mean contact were set to ⟨*u*⟩_H_ = 0.9 and ⟨*u*⟩_L_= 0.1, respectively. Using these criteria, 175 residue-pair contact DOFs were identified for the hBD-3 monomer and 388 were identified for the hBD-3 dimer. PCA was applied to these contact DOFs, and the eight dominant PCs were retained for subsequent analysis. LDA was then applied to these PCs to discriminate between frames corresponding to low and high salt conditions, wild-type and analog forms, and different lipid membrane environments, thereby elucidating the residue-pair contact patterns contributing to these states.

Different from the PCA method described above, which is an unsupervised approach designed to identify the primary sources of variance in the data, LDA is a supervised learning method. In this study, LDA was performed on the truncated conformational space defined by the top eight PCs, yielding linear discriminant (LD) loadings and LD scores. The scores of observations belonging to distinct classes are assumed to follow approximately normal distributions with minimal overlap between the classes. Introducing additional classes requires the inclusion of additional linear discriminants. Specifically, in this case, the linear transformation is defined as LD*_i_* = ∑*_j_* PC*_j_* × C_*i*_^*j*^, where *i*, *j* = 1,...,8 and LD*_i_* is the *i*th LDA score, and PC*_j_* is the *j*th PC projection coordinate. Here, *C*_*i*_^*j*^ denotes the coefficients of this transformation. Note that the LD loading can also be arranged in matrix form and be expressed in a contact heatmap format. Specifically, we define the matrix *d*_*ij*_^(*k*)^ as the *k*th normalized PC loading, and the quantity ∑_*k*_ *C*_*l*_^*k*^ × *d*_*ij*_^(*k*)^ corresponds to the *l*th LD loading.

In order to clearly label the simulation systems in the PCA and LDA figures, abbreviations for system names are given in Table S1.

## 3 Results

### 3.1 Structure and Dynamics Result from Conventional Trajectory Analysis

#### 3.1.1 RMSD, *R*_g_, Number of Hydrogen Bonds Formed, and Shifting of Protein COM

Based on 28 unconstrained μs-long explicit solvent and lipid MD simulation trajectories for both hBD-3 monomers and dimers in wild-type and analog forms, the protein RMSD was calculated after aligning the trajectory to its corresponding initial structure (time-dependent results are shown in Figures S2 and S3 in SI). The hBD-3 monomers reached equilibrium after approximately 500 ns of all-atom MD simulations, whereas equilibration required approximately 1,000 ns for the dimer form. Therefore, in this study, only the second half of the simulation trajectories for both hBD-3 monomers and dimers was used to calculate the average RMSD and *R*_g_, while the full trajectories were used to calculate the number of water translocation events. The average RMSD values and standard deviations for both monomers and dimers are summarized in Table S2. The RMSDs of monomers (3.47∼10.9 Å) are comparable to, but generally slightly smaller than, those of dimers (4.25∼10.42 Å) and are not significantly affected by salt concentration (0.15 M vs 0.30 M) or the lipid chemical composition. In contrast, hBD-3 dimers in the analog form exhibit higher RMSD values than dimers in the wild-type form, as well as monomers in both wild-type and analog forms. On average, the dimers exhibit larger *R*_g_ values than the monomers, independent of salt concentration or lipid chemical composition. For the hBD-3 dimers in the analog form embedded in four types of model bacterial membranes, the *R*_g_ values range from 13.0 to 17.0 Å, whereas those of the monomers range from 10.0 to 13.0 Å.

Analysis of hydrogen bonds formed between hBD-3 and the lipid membranes (results shown in Table S2) indicates that hBD-3 dimers form more hydrogen bonds with the membrane than monomers across all systems. The largest number of hydrogen bonds is observed in the POPC+10%PIP2 system, whereas similar numbers are observed in POPE or POPC mixed with POPG lipid membrane systems. This behavior is potentially attributed to the higher negative charge density of PIP2 compared with POPG, and the higher positive charge of the hBD-3 dimer compared with the hBD-3 monomer.

Analysis of the displacement of the protein center of mass (COM) relative to the lipid bilayer COM (Table S2) shows that hBD-3 dimers and monomers remain stably positioned near the center of the lipid bilayer, with occasional displacements of up to 13.81 Å except for hBD-3 monomer in wild-type and analog forms in the POPE/POPG = 3/1 membrane (initially aligned in Z-orientation) and at high salt concentration (0.3 M). In this condition, hBD-3 monomer has a COM shifting up to 22∼24 Å, as shown in Table S2 (highlighted in red), which corresponds to the shifting of hBD-3 monomer from the center of membrane to the membrane surface. Interestingly, at the physiological salt concentration (0.15 M), the hBD-3 monomers initially aligned in the same orientation can be embedded stably in the same membrane (POPE/POPG = 3/1). This result emphasizes the effect of a high salt concentration on the dynamics of hBD-3 embedded in model membranes. Overall, the hBD-3 dimer can stay more stably in the lipid bilayer than the monomer; and the model G+ bacterial membrane (POPE/POPG = 1:3) is more effective at retaining the hBD-3 at the membrane center than the G- bacterial membrane (POPE/POPG = 3/1), while the POPC+10%PIP2 membrane exhibits intermediate behavior. This trend is consistent with the ranking of the electrostatic interaction between positively charged hBD-3 and the overall negatively charged lipid bilayer. The charge density per lipid is −75% for the G+ bacterial membrane, −25% for the G- membrane and the POPC/POPG = 3:1 membrane, and −40% for the POPC+10%PIP2 membrane; and the charge density of hBD-3 dimer is twice that of the hBD-3 monomer. Thus, the stronger electrostatic interactions between hBD-3 and the membrane correlate with increased stability of the protein within the bilayer over the μs-long simulations, and such an effect is more significant for hBD-3 dimer than hBD-3 monomer. A representative video showing an hBD-3 monomer migrating from the bilayer center to the membrane surface is provided as Video S1 in SI.

#### 3.1.2 Initial and Final Structures, Pore Size Analysis, Lipid Aggregation, and Water Translocation

After μs-long simulations, the final structures of the hBD-3 dimers embedded in different model membranes were extracted and compared with their initial structures (examples are shown in Figure S4). Initially, the hBD-3 dimer forms a tightly bound complex. During the simulations, however, they can form a pore at the center of the membrane, which allows water molecules to stay around the hBD-3 dimer. This pore also facilitates water permeation across the membrane, as water molecules can avoid the hydrophobic lipid tail region at the membrane center and instead traverse the bilayer through the pore/channel. This behavior is illustrated by the side-view structure of the hBD-3 dimer in the analog form embedded in the POPC/POPG = 3:1 membrane shown in Figure 1(a) (water and ions not shown), as well as by the water flow through the hBD-3 dimer depicted in Figure 1(b) (membrane not shown), where blue arrows indicate the direction of water permeation through the pore. In contrast, at the high salt concentration and in G- membrane systems, the hBD-3 monomer initially aligned in Z-orientation migrates out of the membrane and binds to the membrane surface, consistent with the protein COM displacement results discussed above. Together, these observations highlight the effect of a high salt concentration on the dynamic behavior of hBD-3 in lipid membranes.

**Figure 1:**
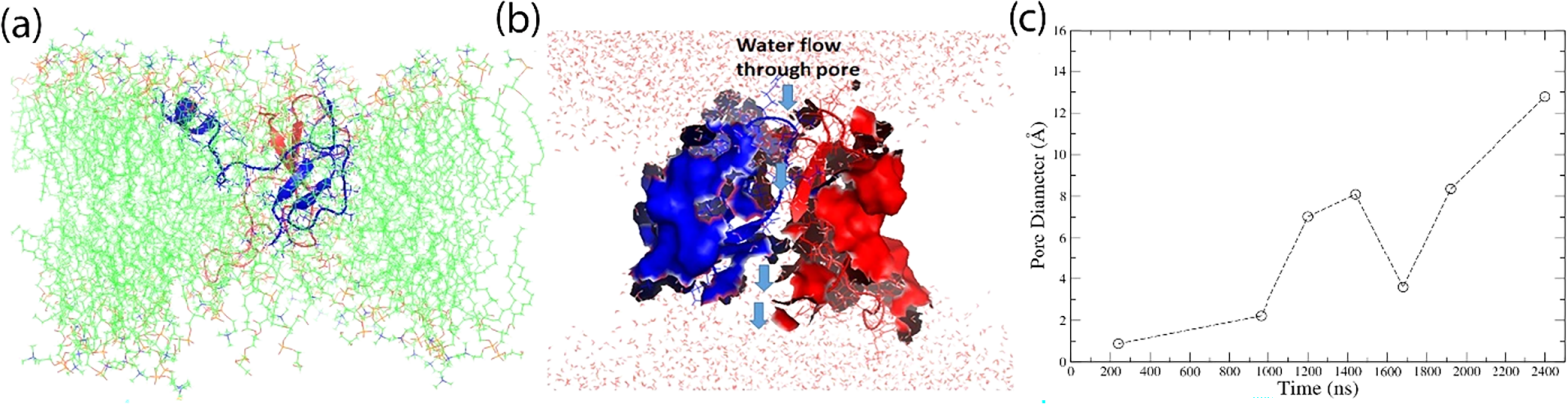
Side view of hBD-3 dimer (the first hBD-3 unit in the dimer shown in red cartoon and the second unit in blue) embedded inside POPC/POPG = 3/1 mixed lipid bilayer after 2.4 μs Anton simulation, which does not show water or ions (a); the water molecules (red dots) around and flowing through the pore formed by hBD-3 dimer (shown in surface with one unit in red and another unit in blue) embedded in the POPC/POPG = 3/1 mixed bilayer system in side view without showing the lipid bilayer (b); and the diameter of the pore formed during 2.4 μs simulations (c).

The pore size formed within the hBD-3 dimer was calculated using the online PoreWalker program,^46^ and the results are shown in Figure 1(c). The pore diameter increased over the course of the 2.4 μs simulations. This increase in dimer pore size is generally consistent with the observed increase in *R*_g_ during the simulation (as indicated by the RMSD, *R*_g_, and the initial and final structures of the hBD-3 dimer shown in Figure S3).

Because hBD-3 is highly positively charged, whereas POPG and PIP2 lipids carry charges of −1e and −4e, respectively, the distribution of negatively charged lipids around hBD-3 was analyzed. Aggregation of negatively charged lipids toward hBD-3 was observed, as shown by the distance histograms of POPG lipids relative to the protein in Figure S5­(a) and of PIP2 lipids relative to the hBD-3 dimer in Figure S5­(b). Similar aggregation behavior was observed in both normal and high NaCl concentration systems over long-term MD simulations. In contrast, such aggregation was not evident in hBD-3 monomer systems or in POPE/POPG = 3:1 lipid bilayer systems.

Although lipid membranes are generally expected to block the transfer of ions and water, the presence of a strongly positively charged peptide monomer or dimer embedded in the membrane can facilitate water permeation across the bilayer as one example structure shown in Figure 1(b). The number of water molecules crossing the lipid bilayer for different systems during the simulations was calculated and is shown in Figure S6­(a),(b) for G+ and G- membranes, respectively. Overall, water molecules cross the model G+ bacterial membrane much faster than the model G- bacterial membrane, and the permeation rate is generally higher in hBD-3 dimer systems than in monomer systems. This behavior likely reflects the stronger electrostatic interactions between hBD-3 dimers and G+ bacterial membranes compared with G- membranes. However, no consistent dependence of the water permeation rate on salt concentration was observed.

### 3.2 Conformational Dynamics from the Residue–Residue Contact Viewpoint

Based on the hypothesis that certain allosteric and other conformational switching mechanisms can be captured through the propagation of collective motions arising from a subset of dynamic residue-level contact changes (formation and dissociation), the present work extends this concept to protein–lipid contacts (implicitly) and explores the benefits of long-term all-atom descriptions of protein dynamics using contact-based statistical analysis.

#### 3.2.1 Contact PC Projections Express Overall Structural Variations Using Collective Coordinates

In order to reduce the number of variables considered in the hBD-3 monomer and dimer systems, PCA was performed by using residue–residue contact information collected over the simulation time. It was found that the first eight PCs account for 50.8% of the total variance in the monomer system (with variances of the top 50 PCs shown in Figure S7) and 45.6% of the total variance in the dimer system (with variances of the top 50 PCs shown in Figure S8). Therefore, focusing on the top eight PCs is reasonable for describing the hBD-3 monomer and dimer systems. This truncated representation of contact dynamics filters out small-amplitude noisy motions captured by higher-order PCs while preserving the majority of the protein’s conformational dynamics.

Projections onto the top two PCs (PC1 versus PC2) were calculated based on the contact dynamics of hBD-3 monomers and dimers under different conditions. The resulting projections are shown in Figure 2(a) for the hBD-3 monomer and in Figure 2(b) for the hBD-3 dimer, where each point represents a specific conformation sampled during the long-term simulations.

**Figure 2:**
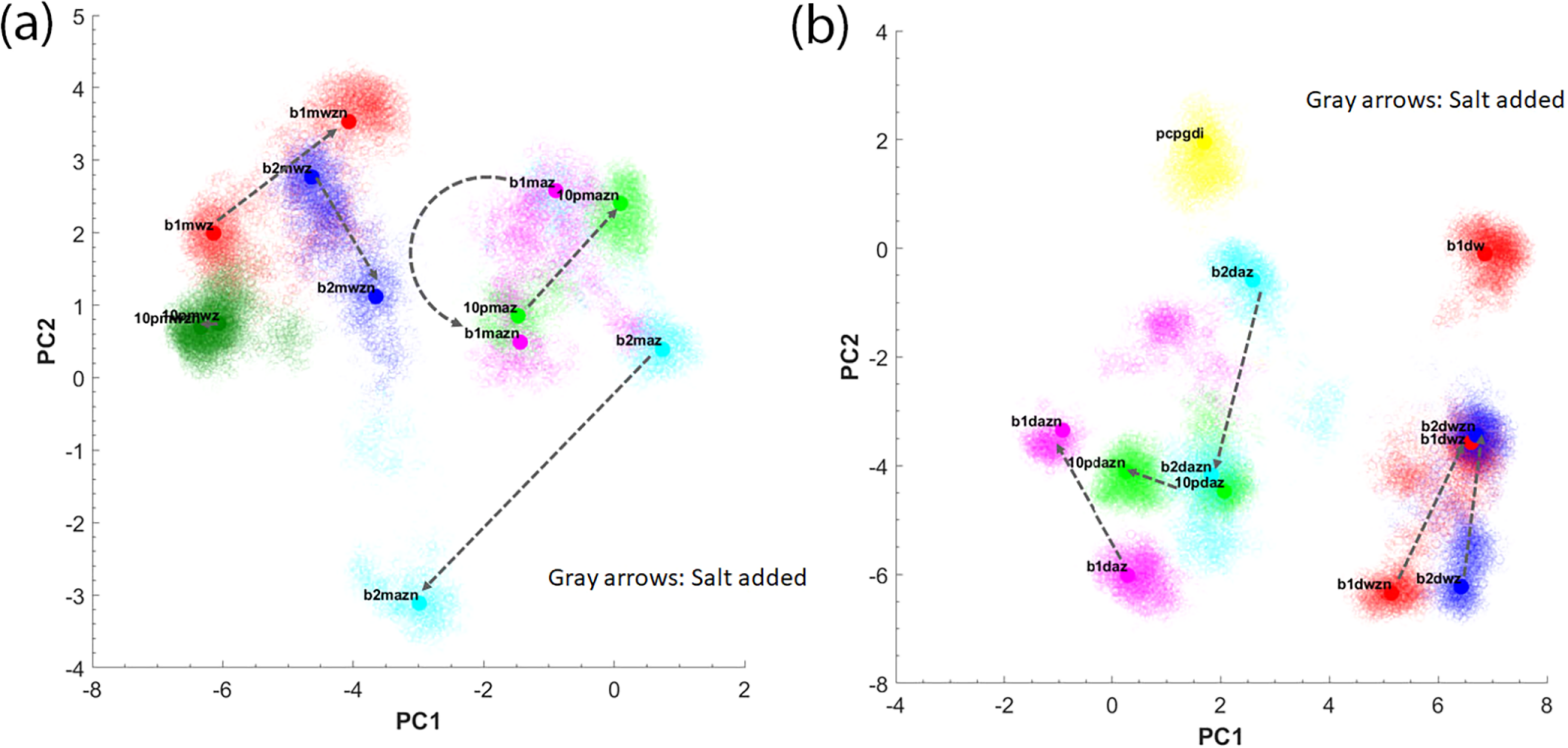
Projection of the top two PCs based on residue–residue contacts of hBD-3 monomer (a) and hBD-3 dimer (b) embedded inside different lipid bilayers, at different salt concentrations, and at different redox conditions. The G+ membrane (abbreviated as b1) results are shown in red or magenta dots, G- membrane (abbreviated as b2) results are shown in blue or cyan dots, while POPC+10%PIP2 membrane results are shown in light green or dark green dots. The dashed lines point from the 0.15 M salt concentration result to the 0.3 M salt concentration result. The gray arrows point from the normal to the high salt concentration systems.

Figure 2(a) shows that the wild-type hBD-3 monomer (conformations of hBD-3 monomer in wild-type in different simulations) exhibits a narrow PC1 projection distribution (with PC1 values mostly below −3.0), reflecting its comparatively rigid structure, whereas the analog monomer (conformations of hBD-3 monomer in analog form in different simulations) displays a broader distribution, with PC1 values mostly above −3.0. Thus, PC1 primarily captures the conformational difference between the wild-type and analog forms. In contrast, the addition of salt (as pointed out by gray arrows) often induces changes along PC2, although not in a consistent direction. This observation suggests that electrostatic interactions strongly influence protein conformations and that membrane chemistry is intimately involved in the structural dynamics of hBD-3.

Figure 2(b) shows that the hBD-3 dimer in the analog form lies on the negative side of PC1, whereas the wild-type dimer lies on the positive side of PC1. Thus, PC1 represents the primary difference between the wild-type and analog forms (folding mode). The POPC+POPG membrane system exhibits higher PC2 values than the other systems; however, PC2 does not clearly distinguish between different lipid compositions or salt concentrations.

#### 3.2.2 Contact PC Loadings (Dynamic Modes) Reveal Structural Fluctuations at the Residue Resolution

To study the dynamic motions using residue–residue contact matrices, the top dynamical mode (eigenvector PC1) is displayed as a contact displacement matrix with the mapped secondary structure in Figure 3(a) (2D contact map view) and as the corresponding 3D cylinder representation in Figure 3(b). Figure 3(a),(b) shows that the first 13 residues, residues 28–30, and the last 3 residues form strong contact interactions, whereas most other regions break contacts, and vice versa. This motion is also illustrated using color-coded cylinders in the right panel, in which blue cylinders represent breaking contacts and red cylinders represent forming contacts. Thus, the major conformational variance described by PC1 corresponds primarily to the expansion of the hBD-3 monomer within the lipid membrane during the μs-long simulations.

**Figure 3:**
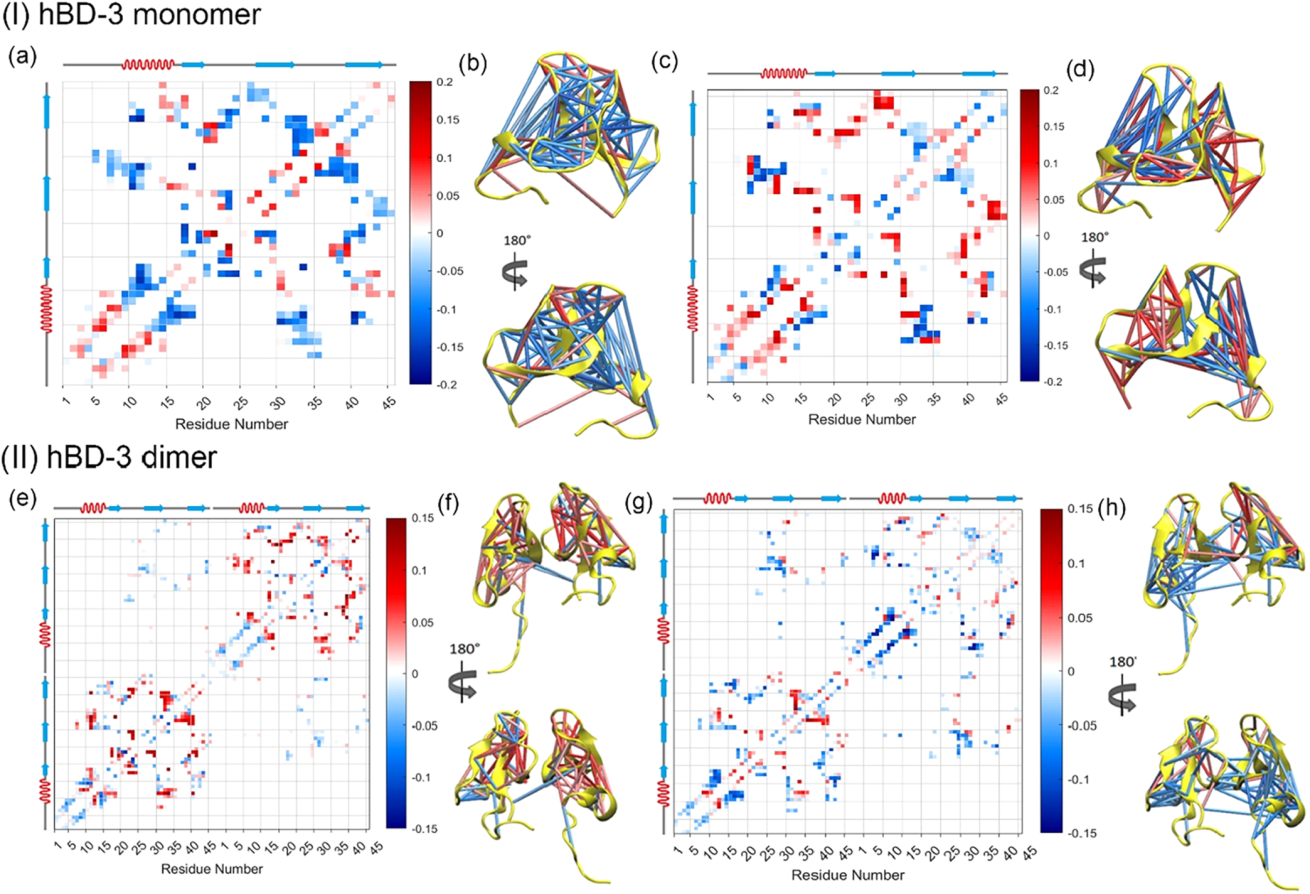
Residue–residue contact matrices for hBD-3 monomer (I) and dimer (II), which are the eigenvector PC1 (a, e) for residue–residue contact and the dynamics information (as colored cylinders) mapped onto the 3D structure (b, f); the residue–residue contact displacement matrices for PC2 (c, g) with the color map shown on its right and the secondary structure of hBD-3 shown on its left and top; and the mapped structure of hBD-3 in front view and back view (d, h). The blue lines represent breaking of contacts, while red lines represent formation of contacts.

Similarly, the contact displacement matrices for PC2 and the corresponding mapped structural representations are shown in Figures 3(c),(d). In this mode, some regions of the hBD-3 monomer form contacts, while others break contacts, suggesting that PC2 represents more complex internal dynamics.

For the hBD-3 dimer, the contact displacement matrices for PC1 and PC2 were calculated and mapped onto the secondary structure, as shown in Figure 3(e)–(h), respectively. Figure 3(e),3(f) shows that PC1 is characterized by dimer dissociation, the formation of contacts within individual hBD-3 units or with surrounding lipids, and overall structural loss, which is expected from long-term sampling in the simulations. Because the analog form lies on the negative side of PC1 (as shown in Figure 2(b)), the majority of contacts are red, indicating contact formation as hBD-3 transitions to the wild-type (oxidized) state. In contrast, Figure 3(g),(h) shows that PC2 represents the formation of contacts between hBD-3 units and lipids, accompanied by the breaking of some local contacts within the hBD-3 units.

#### 3.2.3 Physical and Chemical Factors Affecting Structural Dynamics Revealed by Contact LDA

In the previous subsections, the structural fluctuations were characterized using PCA, which identifies the dominant modes of contact variation. However, some physical factors (such as disulfide bond constraints) and chemical factors (such as lipid composition) were not explicitly labeled in this unsupervised analysis. Here, we focus on using LDA to explicitly compare how different factors affect the structural dynamics.

##### 3.2.3.1 Effect of Salt Concentration

In order to separate the conformational distributions of the hBD-3 monomer under different salt concentrations, redox conditions, and lipid membrane environments, LDA was conducted by using the collective coordinates (PC projections) obtained from the contact PCA. Residue–residue contact maps were generated from the LDA results for the hBD-3 monomer along with the corresponding mapped structure representations. The probability density of LD1_salt_ is shown in Figure 4(a), the contact matrix of LD1_salt_ in Figure 4(b), and the secondary structure with mapped contacts in Figure 4(c) for different salt concentrations.

**Figure 4:**
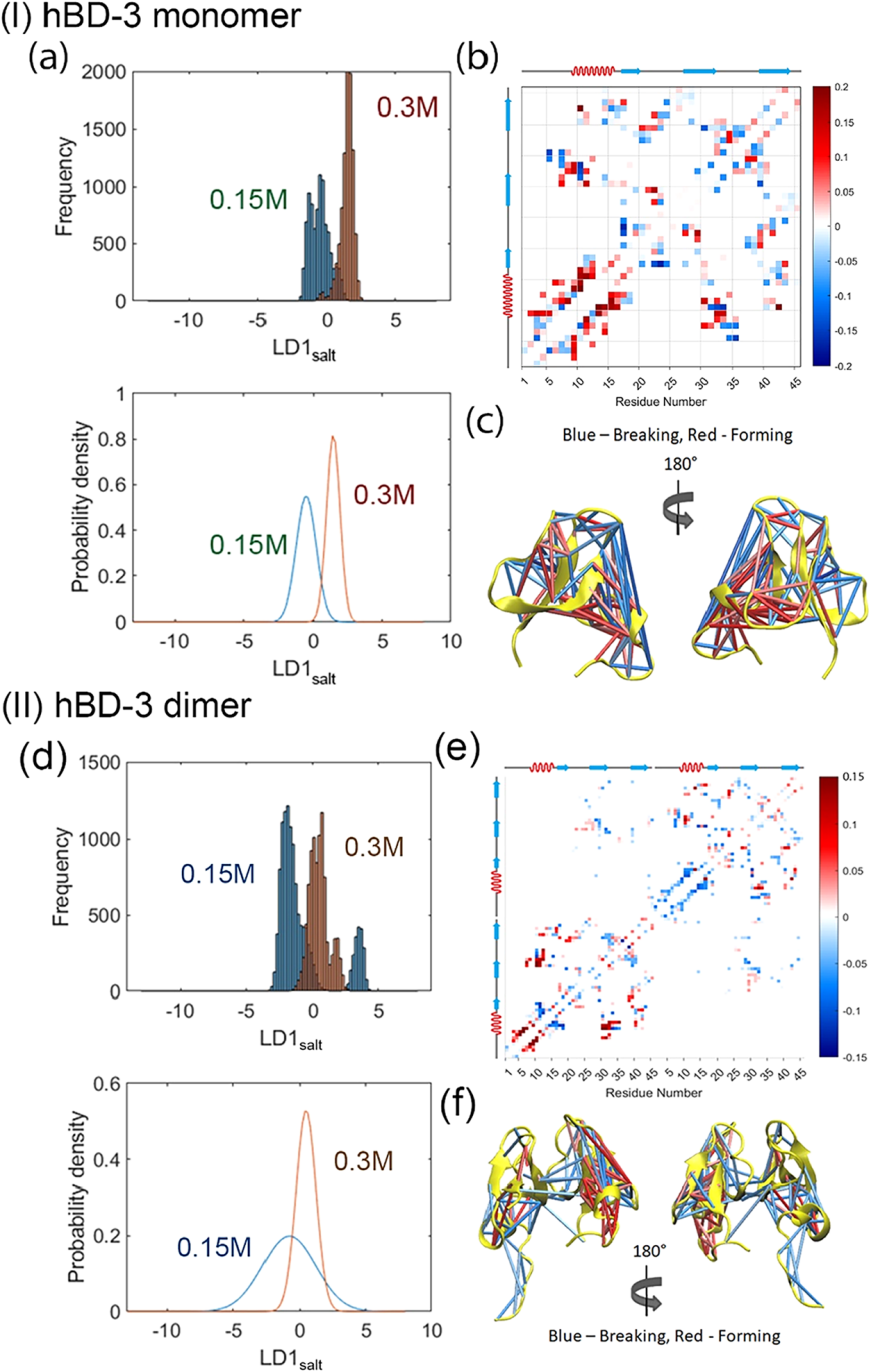
LDA scores of hBD-3 monomer (I) and dimer (II) at different salt concentrations (a, d), the mean residue–residue contact matrix (b, e), which describes the first linear discriminant, LDA1 with respect to salt concentration (LD1_salt_), and the secondary structure mapped contact displacement matrices (c, f) for hBD-3 at different salt concentrations. The blue color represents breaking contacts, while the red color represents forming contacts upon increasing salt concentration.

Figure 4(a) shows that LD1_salt_ effectively separates the conformational distributions at normal (0.15 M) and high (0.3 M) salt concentrations with minimal overlap. As the salt concentration increases from 0.15 to 0.3 M, the LD1_salt_ increases accordingly. Figure 4(b) shows the residue–residue contact matrix corresponding to LD1_salt_. With increasing salt concentration, the N-terminal region of hBD-3 tends to form more local contacts and additional contacts with the β2 sheet, while other regions undergo rearrangements, as indicated by the predominantly blue regions in the contact matrix. Figure 4(c) further indicates that with increasing salt concentration, the central region of hBD-3 breaks contacts, whereas peripheral regions form additional contacts with lipids. Together, these results suggest that the hBD-3 monomer expands its structure in the lipid membrane as the salt concentration increases.

In contrast, residue–residue contact matrices for the hBD-3 dimer in both wild-type and analog forms at different salt concentrations are shown in Figure 4(d)–(f), respectively. These figures show that the conformational distributions of the hBD-3 dimer at different salt concentrations are not clearly separable. Instead, changes in salt concentration appear to contribute to dimer dissociation while promoting the formation of local contacts within individual hBD-3 subunits.

##### 3.2.3.2 Effect of Redox Conditions

Similarly, based on the conformations of hBD-3 monomer under different redox conditions, residue–residue contact displacement matrices were calculated, and these contacts were mapped onto the secondary structure of hBD-3 monomer. The results are shown in Figure 5(a)–(c). Because the wild-type state is located on the left side of the analog state on LD1_redox_, and the majority of the contact matrix is in blue, the loss of structure-stabilizing disulfide bonds leads to significant destabilization of the wild-type structure, resulting in the breaking of most central contacts in hBD-3.

**Figure 5:**
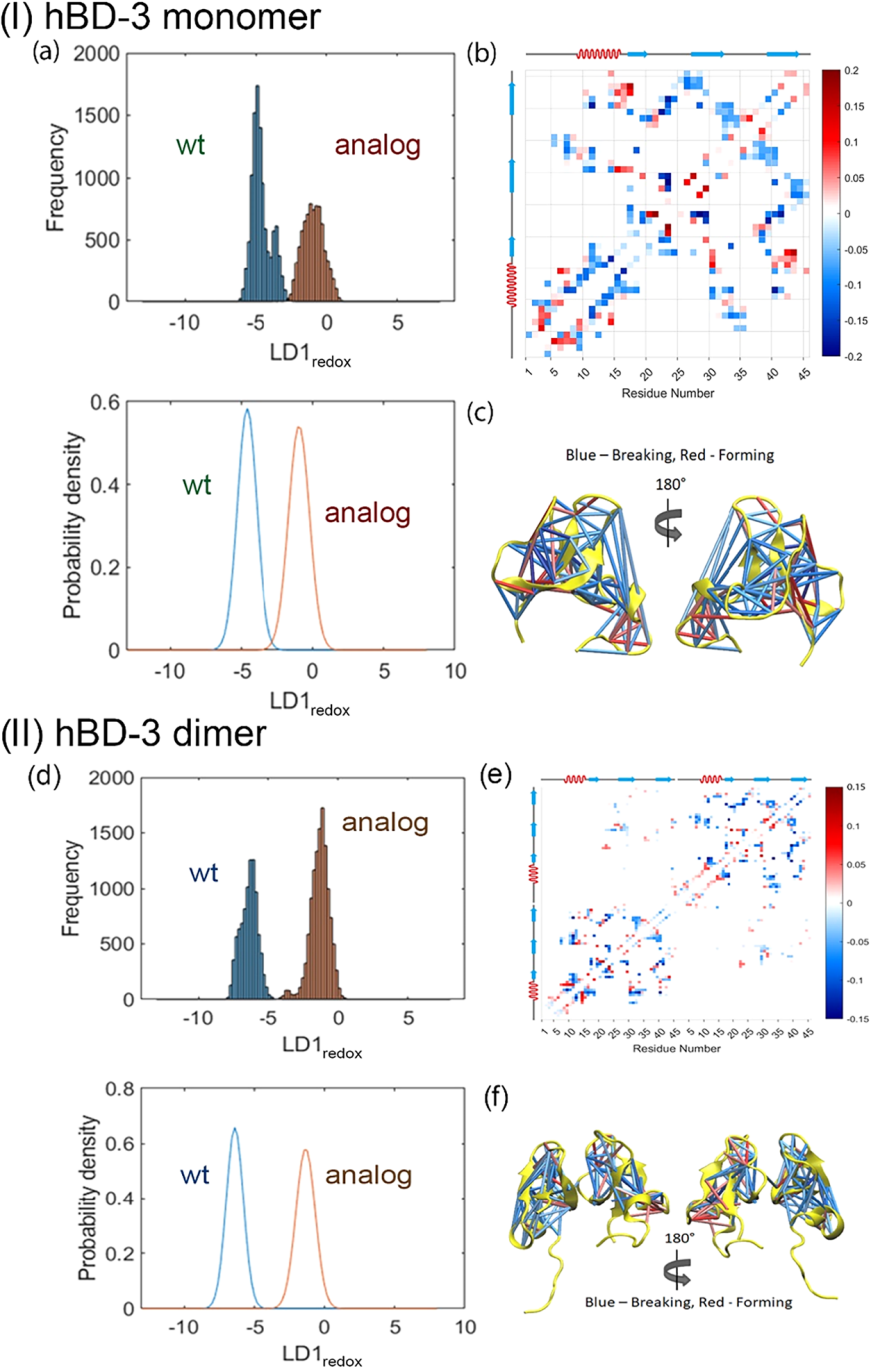
LDA scores of hBD-3 monomer (I) and dimer (II) at different redox conditions (a, d), the mean residue–residue contact matrix of LD1_redox_ (b, e) which describes the first linear discriminant with respect to redox conditions, and the secondary structure mapped with contact matrices (c, f) for hBD-3 at different redox conditions. The blue color represents breaking contacts, while the red color represents forming contacts upon the reduced state perturbation.

Figure 5(d)–(f) shows that LDA can separate the contact conformations of the hBD-3 dimer under different redox conditions. The wild-type hBD-3 dimer is distributed on the left side of the analog dimer along LDA1. The majority of the contact matrix is blue, corresponding to the contact breaking; thus, the disruption of disulfide bonds reinforces the loss of contacts in hBD-3. Different redox conditions contribute to the breaking of intramolecular contacts within each hBD-3 unit in the dimer, while local contacts within the hBD-3 molecules exhibit more complex dynamics, potentially modulated by salt effects or by lipid membranes.

##### 3.2.3.3 Effect of Membrane Chemical Composition

To examine the effect of lipid chemical composition on the contact maps of hBD-3 monomer, LDA scores for the hBD-3 monomer in different lipid membranes (LDA_membrane_) are shown in Figure 6(a), with the corresponding residue–residue contact displacement matrices and secondary structure mapped contacts shown in Figure 6(b)–(e). Figure 6(a) indicates that LDA with respect to lipid composition does not reveal a clear or systematic dependence, in contrast to the effects observed for salt concentration and redox conditions in Figures 4(a) and 5(a), where the minimal overlap of the probability distributions suggests that contact degrees of freedom are strongly affected by salt concentration and redox conditions.

**Figure 6:**
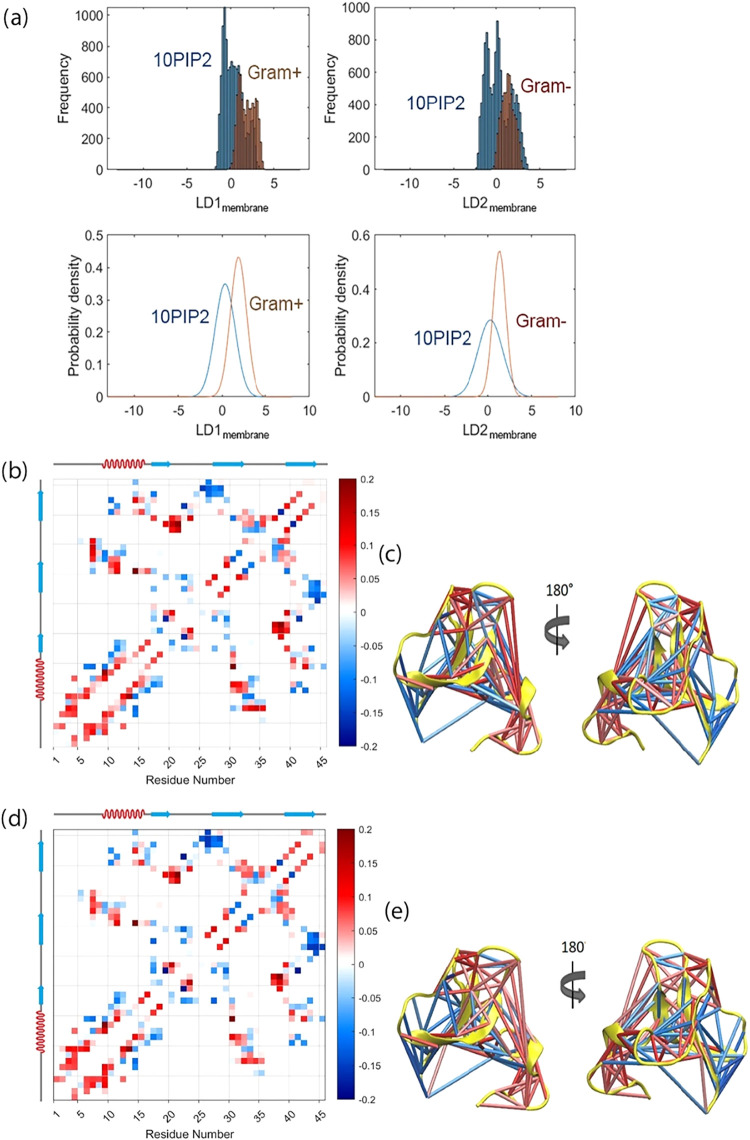
LDA scores for the hBD-3 monomer embedded inside different lipid bilayers (a). The mean residue–residue contact matrix of LD1_membrane_ (b), which describes the first linear discriminant with respect to the lipid chemical composition conditions from POPC+10%PIP2 to G+ membranes, and the secondary structure mapped with contact matrices (c) for hBD-3 monomer at different lipid chemical compositions. The mean residue–residue contact matrix of LD2_membrane_ (d) describes the first linear discriminant with respect to the lipid chemical composition conditions from POPC+10%PIP2 to G- membranes and the secondary structure mapped with contact matrices (e) for hBD-3 monomer at different lipid chemical compositions. The blue color represents breaking contacts, while the red color represents forming contacts.

LD2_membrane_ is very similar to LD1_membrane_. As the membrane composition changes from POPC+10%PIP2 to G+ or G- membranes, the N-terminal region of hBD-3 tends to shift from the β2 sheet toward the adjacent loop region. The C-terminal tail, which contains several positively charged residues, also moves away from the core of the protein. More strongly negatively charged membranes appear to induce a slightly more compact overall structure of hBD-3, which is accompanied by subtle rearrangements.

Similarly, to examine the effect of lipid chemical composition on the contact maps of the hBD-3 dimer, the LDA scores of the hBD-3 dimer in different lipid membranes (LDA_membrane_) are shown in Figure 7(a), with the corresponding residue–residue contact displacement matrices and secondary structure mapped contacts shown in Figure 7(b)–(e).

**Figure 7:**
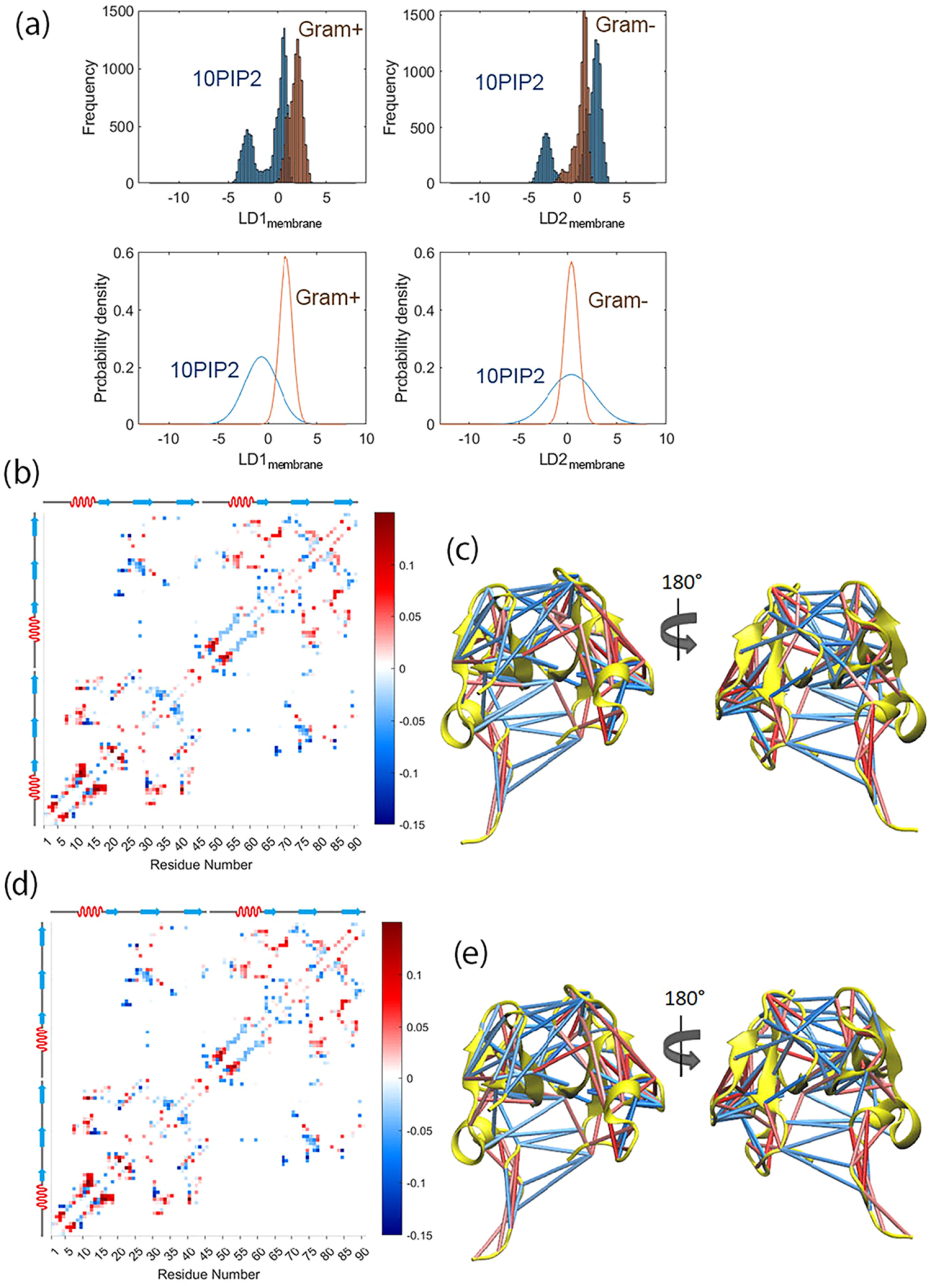
LDA scores for hBD-3 dimer embedded inside different lipid bilayers (a). The mean residue–residue contact matrix of LD1_membrane_ (b), which describes the first linear discriminant with respect to the lipid chemical composition conditions from POPC+10PIP2 to G+ membranes, and the secondary structure mapped with contact matrices (c) for hBD-3 dimer at different lipid chemical compositions. The mean residue–residue contact matrix of LD2_membrane_ (d), which describes the first linear discriminant with respect to the lipid chemical composition conditions from POPC+10%PIP2 to G- membranes, and the secondary structure mapped with contact matrices (e) for hBD-3 dimer at different lipid chemical compositions. The blue color represents breaking contacts, while the red color represents forming contacts.

Figure 7(a) shows that although the LD2_membrane_ cannot separate the effects of POPC+10%PIP2 and G- membranes, it can distinguish between POPC+10%PIP2 and G+ membranes. LD1_membrane_ and LD2_membrane_ are nearly identical. Most contact breaking occurs between the two hBD-3 units, suggesting a mild preference of both G+ and G- membranes for dimer dissociation. Within each unit, contacts are predominantly formed.

In contrast, Figures 4(d) and 5(d) show that different contact conformations can be readily separated under different redox conditions, whereas salt concentration does not have a consistent effect on the contact degrees of freedom of hBD-3 dimers. This suggests that dimerization may enhance hBD-3′s resistance to salt effects. Figure 7(a) further indicates that the effects of POPC+10%PIP2 and G+ membranes can be distinguished. Because LD2_membrane_ cannot separate the effects of the G- membrane from those of the POPC+10%PIP2 membrane, the hBD-3 dimer appears capable of distinguishing between G+ and G- membranes. This observation is consistent with experimental findings that hBD-3 functions in a dimer form^29^ and retains antibacterial activity even at high salt concentrations. Also, hBD-3 shows selectivity, disrupting different bacterial membranes.

##### 3.2.3.4 Contact Matrix PCA and LDA Results Summary

In summary, based on contact matrices and the corresponding PCA and LDA of the hBD-3 monomer, PC1 primarily distinguishes between the analog and wild-type forms. LDA relies largely on PC1 and indicates that the analog form experiences significant structural loss due to the absence of stabilizing disulfide bonds. The LDA results further show that the salt concentration has a relatively consistent effect on the contact degrees of freedom of the hBD-3 monomer across different lipid membranes. In contrast, changes in lipid membrane composition have a less pronounced effect on the contact degrees of freedom than redox conditions or salt concentration for this protein.

For the hBD-3 dimer embedded in different lipid membranes, PC1 also primarily distinguishes between the analog and wild-type forms. LDA reveals that hBD-3 dimer structures are not sensitive to salt concentration, which is consistent with the assumption that hBD-3 can function at high salt conditions. The changes in the lipid membrane composition have limited effects on hBD-3 dimer dynamics.

## 4 Discussion

In this work, the dynamics of hBD-3 monomers and dimers embedded in different negatively charged lipid bilayers were investigated using a conformational dynamics analysis method based on μs-long MD simulations. We found that hBD-3 dimers and monomers can remain stably embedded within lipid membranes except the hBD-3 monomer in wild-type and analog forms in model G- membrane and at high salt concentration (0.3 M NaCl). As a comparison, at the normal (physiological) salt concentration (0.15 M), the hBD-3 monomer in wild-type and analog forms initially inserted in the same orientation can stay in the same membrane stably. That emphasized the effect of a high salt concentration on the dynamics of hBD-3 embedded in membranes. As another comparison, the hBD-3 dimer initially in Z-orientation and at high salt concentration can stay inside the model G- membrane stably during 2.4 μs simulations. Thus, the shifting of the hBD-3 monomer from the membrane center to the membrane surface also emphasized the effect of hBD-3 oligomerization on hBD-3 dynamics in the membrane. Since the hBD-3 dimer doubles the positive charge density of the hBD-3 monomer and the G- membrane has the lowest negative charge density out of the four bilayers studied, the hBD-3 monomer and model G- membrane form the weakest electrostatic interaction among all the systems. Since high salt concentration can weaken the electrostatic interaction between hBD-3 and the overall negatively charged membrane, this can trigger the shifting of the hBD-3 monomer from the center of the membrane to the membrane surface. In combination with LDA and secondary structure mapped contact displacement matrices shown in Figure 4(c), which indicates that hBD-3 monomer expands its structure as the salt concentration increases, it is expected that with high salt concentration, hBD-3 changes its structure inside the membrane that facilitates its movement from the hydrophobic lipid bilayer center to the membrane surface.

In addition, in AMP-membrane systems, water molecules can play an important role in the dynamics of AMP, in addition to the direct protein–membrane interactions.^54^ In this study, it was found that water molecules can permeate through hBD-3 dimers via a water channel. In order to analyze the water dynamics, the number of water molecules crossing the lipid bilayer over time was calculated, as shown in Figure S6. It was found that water can translocate through hBD-3 dimer-bilayer systems faster than in hBD-3 monomer-bilayer systems. Also, the stronger the negative charge of lipid bilayer, the stronger the interaction between hBD-3 and lipid membrane, and the faster the water translocation. With the assumption that the larger the pore/channel formed in the hBD-3 membrane system, the faster the water translocation and more fragile the membrane, the G+ membranes which embedded hBD-3 dimer should be more prone to breakage; on the other hand, the hBD-3 monomer in the membrane system should be less prone to breaking. These factors collectively contribute to the distinct dynamical behaviors of hBD-3 monomers and dimers when interacting with model lipid membranes.

In this study, 28 unconstrained all-atom MD simulations were conducted, and each simulation was conducted for at least 1.5 μs. Based on the limited results in this work, some unique insights can be derived for understanding the functional mechanisms of hBD-3.

(1) The Selectivity of hBD-3 Interacting with Different Bacterial Cell Membranes: In this study, the LDA results indicate that the hBD-3 dimer exhibits distinguishably different dynamical behavior in POPC+10%PIP2 and POPE/POPG = 1:3 membranes but similar dynamical behavior in POPE/POPG = 3/1 and POPC+10%PIP2 membranes. Thus, hBD-3 dimer can distinguish G+ membranes from G- membranes. However, such distinctions are not observed for the hBD-3 monomers. So, changes in lipid membrane composition have a negligible effect on the dynamics and interactions of hBD-3 monomers with membranes but moderate effect on the dimers with membranes. The result from the hBD-3 dimer agrees with experimental observation that hBD-3 possesses selectivity when disrupting different cell membranes. hBD-3 can significantly disrupt the inner membrane of G- bacteria, and cross model bacterial membranes represented by POPC/POPG = 1/1 lipid bilayers.^23^ hBD-3 also can bind with lipid II when killing G+ bacterial membrane.^55^ Since forming a dimer increases the charge density of hBD-3 in each system, and there are strong electrostatic interactions between the strongly positively charged hBD-3 and overall negatively charged membranes, this finding indicates that electrostatic interactions play a major role in membrane selectivity, and contribute to the membrane-disrupting capability of hBD-3 in both model G+ and G- membranes, although specific lipids such as lipid II in G+ membranes further enhance antibacterial activity.^55^
(2) hBD-3′s Antibacterial Activity Dependence on Salt: In this study, we found that high salt concentration can weaken the electrostatic interaction between hBD-3 and overall negatively charged lipid bilayer, thus triggering the shifting of hBD-3 monomer (in both wild-type and analog forms) from the center of model G- membrane to the membrane surface but not hBD-3 dimer. Under the influence of salt, local contacts within individual hBD-3 units may form, while contacts between hBD-3 units may break; however, this effect has a limited impact on the overall dynamical characteristics of the hBD-3 dimer. Since hBD-3 can form dimers even at low concentrations (which increases its charge density) and functions in a dimer form,^29^ strong positive charge density of hBD-3 should contribute to the fact that its antibacterial activity is largely insensitive to salt concentration.^27^
(3) The Antibacterial Activity of hBD-3 in Different Redox Conditions: In the LDA, the coefficients associated with *C*_1_^*j*^ are more significant than those of other components, indicating that PC1 makes the dominant contribution to the LDA results. Focusing on PC1, we were able to clearly distinguish the wild-type and analog forms for both hBD-3 monomers and dimers. Our results also show that hBD-3 wild-type and linear analog exhibit distinct dynamical characteristics when embedded in lipid membranes. With the general assumption that protein structure determines its function, our result suggests that the wild-type and analog forms of hBD-3 may translocate through negatively charged membranes via different mechanisms, even though experimental studies have shown that the disulfide bonding status does not significantly affect the antibacterial activity of hBD-3.^56,57^

(4) The Concentration Effect of hBD-3 on Its Membrane Disruption Capability: The hBD-3 monomer and dimer show different dependences on factors considered in this study. The hBD-3 dimer shows no salt-dependence and can distinguish model G+ and G- membranes; while the hBD-3 monomer shows salt-dependence and almost cannot distinguish different negatively charged membranes, although both monomer and dimer have distinguishable conformational dynamics in wild-type and analog forms. Since the activity of the hBD-3 dimer agrees with experimental observations, our result suggests that hBD-3 should mostly function in a dimer form. Since forming a dimer doubles the concentration of hBD-3 in the system, these findings also suggest that only at high concentrations does hBD-3 gain the ability to discriminate between G+ and G- membranes, show insensitivity to salt concentration, and potentially adopt distinct functional mechanisms. Overall, our results highlight the importance of the hBD-3 concentration in modulating its dynamics and biological function. In addition, because of the stronger electrostatic interaction between the hBD-3 dimer and membranes than the hBD-3 monomer, negatively charged lipids such as POPG and PIP2 tend to aggregate around hBD-3, and this effect is more pronounced in hBD-3 dimer systems than in monomer systems.

## 5 Conclusions

μs-long simulations were conducted on hBD-3 monomers and dimers in wild-type and analog forms embedded in four different types of model membranes and under both normal and high salt concentrations. The lipid membranes studied include a model G+ bacterial membrane (represented by a POPG/POPE = 3:1 mixture), a model G- bacterial membrane (represented by a POPG/POPE = 1:3 mixture), a POPC+10%PIP2 mixture, and a POPC/POPG = 3:1 mixture. Trajectory analysis showed that negatively charged lipids such as POPG and PIP2 can aggregate toward the positively charged hBD-3 during μs-long simulations. However, this phenomenon was not pronounced in hBD-3 monomer systems or in POPE/POPG = 3:1 lipid bilayers.

Based on RMSD, COM displacement, and water translocation analyses, hBD-3 in both monomeric and dimeric forms was found to be dynamically active rather than static within negatively charged membranes. The hBD-3 monomers and dimers can stay stably at the center of the lipid bilayers except for the hBD-3 monomer in model G- membrane and at high salt concentration. The hBD-3 dimer can form a pore within the membrane, facilitating the water translocation across the bilayer. Water molecules translocate through the membrane more rapidly in hBD-3 dimer systems than in monomer systems, and translocation occurs faster in model G+ membranes than in G- membranes.

Contact dynamics analysis revealed that PC1 primarily distinguishes between the analog and wild-type forms of hBD-3 in both monomeric and dimeric states. For the hBD-3 monomer, LDA relies mainly on PC1 and indicates that the analog form experiences significant structural loss due to the absence of stabilizing disulfide bonds. The LDA results further show that salt concentration has a relatively consistent effect on the contact degrees of freedom of the hBD-3 monomer across different lipid membranes. In contrast, no clear salt effect was observed for hBD-3 in the dimeric form. Changes in the lipid membrane composition had limited effects on the contact degrees of freedom for both hBD-3 monomers and dimers, although hBD-3 dimer can distinguish G+ from G- membranes.

Overall, these results suggest that wild-type and analog forms of hBD-3 may translocate across negatively charged lipid membranes via different mechanisms, and hBD-3 should function in a dimer form.

## Supplementary Material





## Notes

List of simulations performed in this project, average RMSD, *R*_g_, number of hydrogen bonds formed between protein and membrane, initial structures of hBD-3 in membrane, RMSD of hBD-3 in membrane over simulation time, last structures of simulation systems, number density of POPG/PIP2 in simulations, number of water molecules crossed membranes, and variances of top 50 PCs (PDF)
Migration of hBD-3 monomer from bilayer center to the membrane surface (Video S1) (INDD)

## Notes

**Data and Software Availability statement:** The authors declare that the data supporting the findings of this study are available within the paper and its Supporting Information file.
