# Supplementary material for: Molecular Behavior of Human β Defensin Type 3 Embedded in Different Model Lipid Membranes
Source: J Chem Inf Model. 2026 Apr 30;66(9):5357–71. doi: 10.1021/acs.jcim.5c02937 (PMC13169353; doi:10.1021/acs.jcim.5c02937)
Supplement: Supplementary file 2 [file ci5c02937_si_002.pdf]

Supplemental Information

Molecular Behavior of Human β Defensin Type 3 Embedded in Different Model Lipid Membranes

Jackson Penfield[1], Tongye Shen[2], George R Rucker[1], Liqun Zhang[3,4]

[1] Department of Chemical Engineering, Tennessee Technological University, Cookeville, TN,38505, USA
[2] Department of Biochemistry and Cellular and Molecular Biology, University of Tennessee, Knoxville, TN, 37996, USA
[3] Chemical Engineering department, University of Rhode Island, Kingston, RI, 02881
[4] Department of Chemical Engineering, Hampton University, Hampton, VA, 23669, USA

1. Tables

Table S1. Simulations performed in this project in two different initial insertion orientations (Z direction or head direction) and in different negatively charged lipid bilayers, with the total simulation time, concentration of salt, number of atoms in the simulation box, box size and the job name listed.

Simulations (orientation)	State	Time (ns)	[NaCl] (M)	Number of atoms	Box size (Å^3)	Job name
POPG/POPE=3:1	G+					
Z	m,wt	2400	0.15	63291	83.4x83.4x89.4	b1mwz
Z	m,wt	2400	0.3	63399	81.9x81.9x93.3	b1mwzn
Head	m,wt	2400	0.15	62823	80.1x80.1x96.2	b1mw
Z	m,an	1500	0.15	63189	82.3x82.3x91.9	b1maz
Z	m,an	1500	0.3	63420	82.1x82.1x92.6	b1mazn
Z	d,wt	2400	0.15	63028	83.9x83.9x88.2	b1dwz
Z	d,wt	2400	0.3	63171	83.8x83.8x88.6	b1dwzn
Head	d,wt	2400	0.15	65030	81.6x81.6x96.1	b1dm
Head	d,wt	4800	0.3	58695	81.6x81.6x86.2	b1dmn
Z	d,an	2400	0.15	62941	84.2x84.2x87.4	b1daz
Z	d,an	2400	0.3	62930	83.3x83.3x89.1	b1dazn
POPE/POPG=3:1	G-					
Z	m,wt	1500	0.15	62080	79.3x79.3x97.0	b2mwz
Z	m,wt	1500	0.30	62280	78.6x78.6x99.1	b2mwzn
Z	m,an	1500	0.15	62140	78.6x78.6x98.6	b2maz
Z	m,an	1500	0.30	62324	79.4x79.4x97.1	b2mazn
Z	d,wt	1500	0.15	62120	81.8x81.8x90.9	b2dwz
Z	d,wt	1500	0.30	62210	82.9x82.9x88.7	b2dwzn
Z	d,an	1500	0.15	62040	81.0x81.0x92.7	b2daz
Z	d,an	1500	0.30	62181	81.0x81.0x93.3	b2dazn

POPC/POPG=3:1						
Z	d,an	2400	0.15	49294	72.0x72.0x92.9	Pcpgdi
POPC+10%PIP2						
Z	m,wt	2400	0.15	67870	83.6x83.6x94.3	10pmwz
Z	m,wt	2400	0.30	68277	81.6x81.6x99.8	10pmwzn
Z	m,an	1500	0.15	67936	82.7x82.7x96.4	10pmaz
Z	m,an	1500	0.30	68182	83.8x83.8x94.4	10pmazn
Z	d,wt	2400	0.15	67791	84.2x84.2x92.9	10pdwz
Z	d,wt	1500	0.30	67903	82.8x82.8x96.2	10pdwzn
Z	d,an	1500	0.15	67560	84.9x84.9x91.0	10pdaz
Z	d,an	2400	0.30	67560	84.5x84.5x91.7	10pdazn

Note of abbreviations in state and job names: m is the abbreviation of monomer; d is for dimer; wt is for wildtype, an is for analog. G+ is for Gram-positive bacterial membrane represented by lipid mixture having POPG/POPG=3/1, and G- is the abbreviation of Gram-negative bacterial membrane represented by lipid mixture having POPE/POPG=3/1.

Table S2: The average RMSD, R_g, number of hydrogen bonds (HBonds) formed between the protein and the lipid bilayer, and the shifting distance of protein COM relative to the COM of lipid bilayer. Note of abbreviations in simulation descriptions: "m" is the abbreviation of monomer; "d" is for dimer; "wt" is for wildtype, "an" is for analog; "Z" means initial orientation aligned along Z-axis as shown in Figure S1(Left); Head indicates that the initial structure aligned in head orientation as shown in Figure S1(Middle) and (Right).

Simulations (orientation)	[NaCl](M)	RMSD (Å)	R_g (Å)	Hbonds	COM shifting (Å)
POPG/POPE=3/1					
Z,m,wt	0.15	6.54±0.4	11.1±0.4	14.54 ±3.4	10.03 ±2.2
Z,m,wt	0.30	4.74±0.6	10.6±0.2	12.14±3.8	13.81±2.2
Head,m,wt	0.15	5.50±1.3	11.40±0.4	13.28±3.3	7.31±1.6
Z,m,an	0.15	10.9±0.3	11.1±0.4	14.5±3.5	-7.10±1.5
Z,m,an	0.30	5.72±1.2	11.0±0.4	15.1±3.5	9.97±1.7
Z,d,wt	0.15	5.69±0.8	14.30±0.4	23.80±4.6	-1.51±1.4
Z,d,wt	0.30	4.25±0.3	13.3±0.2	23.8±4.6	-1.78±1.6
Head,d,wt	0.15	5.71±0.4	14.08±0.4	26.33±4.7	-0.4±1.3
Head,d,wt	0.30	10.42±0.5	15.03±0.2	25.96±4.6	1.11±1.4
Z,d,an	0.15	6.98±0.4	13.48±0.3	24.96±4.6	-0.1±1.3
Z,d,an	0.30	4.74±0.4	13.96±0.4	24.94±4.9	-1.9±1.7
POPG/POPE=1/3					
Z,m,wt	0.15	4.26±0.5	10.9±0.3	13.29±3.4	-7.17±2.4
Z,m,wt	0.30	4.19±0.7	10.4±0.4	12.23±3.2	22.88±2.1
Z,m,an	0.15	9.76±0.6	12.0±0.5	15.21±3.6	11.76±2.5
Z,m,an	0.30	3.69±0.4	10.64±0.3	11.86±3.1	24.71±1.6
Z,d,wt	0.15	9.20±0.9	14.78±0.4	24.08±4.9	-9.43±1.6
Z,d,wt	0.30	5.38±0.4	13.3±0.4	23.50±4.6	-1.80±1.8
Z,d,an	0.15	6.71±0.5	15.15±0.4	26.09±4.8	10.35±2.1

Z,d,an	0.30	8.21±0.7	16.54±0.6	20.89±4.4	-7.15±1.4
POPC/POPG=3/1					
Z,d,an	0.15	7.10±0.6	15.1±0.5	23.96±5.0	3.32±1.4
POPC+10%PIP2					
Z,m,wt	0.15	3.47±1.0	11.11±0.3	14.97±3.4	3.72±2.2
Z,m,wt	0.30	4.05±0.4	11.78±0.4	9.37±3.5	18.10±1.7
Z,m,an	0.15	5.92±0.5	12.43±0.4	19.46±3.6	7.12±1.3
Z,m,an	0.30	5.17±0.6	10.08±0.3	12.49±3.2	-2.60±1.8
Z,d,wt	0.15	5.15±1.0	14.3±0.5	28.14±4.5	-10.01±1.2
Z,d,wt	0.30	5.14±1.0	14.3±0.6	23.61±5.0	-7.70±1.1
Z,d,an	0.15	6.16±1.0	14.2±0.4	31.18±4.9	-1.72±1.8
Z,d,an	0.30	5.35±1.0	13.9±0.3	30.98±4.7	4.05±1.0

2. Figures

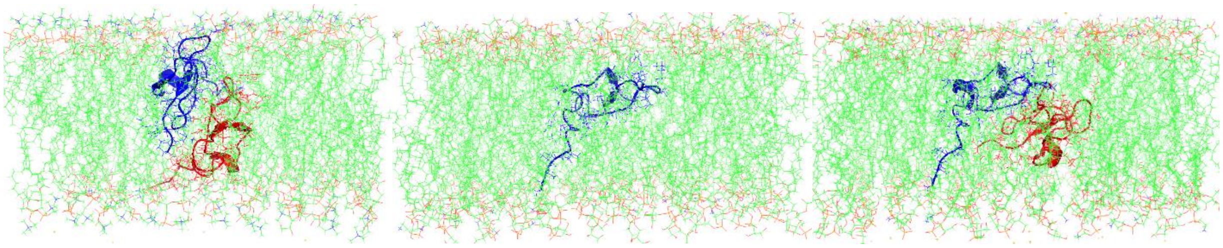

Figure S1. Initial structures of the hBD-3 dimer inside the POPC+POPG mixed lipid bilayers (Left), and hBD-3 monomer (Middle) and dimer (Right) inside the G+ bacterial lipid membrane. The initial orientation of dimer in (Left) is Z direction, while the initial orientations of monomer and dimer in (Middle) and (Right) are head direction. The initial structures in G-, and POPC+10%PIP2 lipid bilayers are aligned either in head orientation or Z-direction in this project.

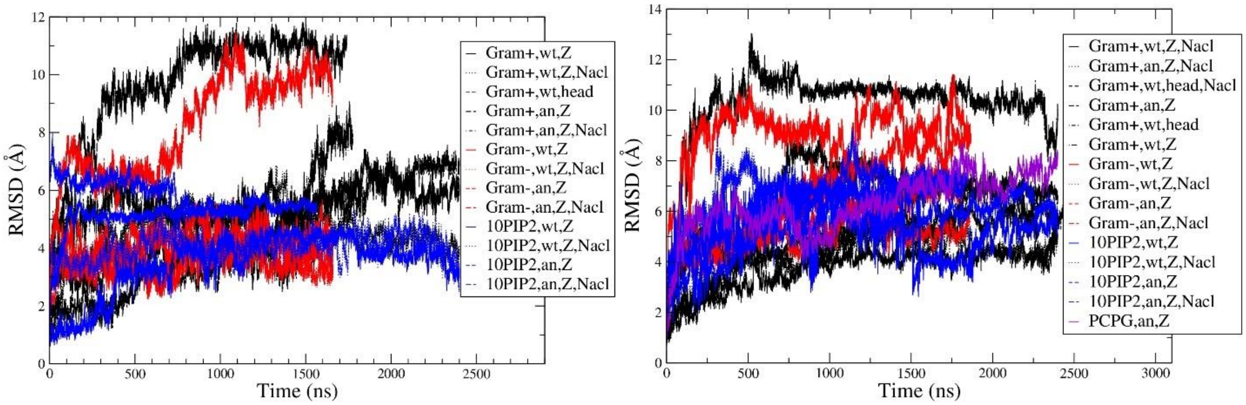

Figure S2. Comparison of RMSD of hBD-3 monomer (Left), and hBD-3 dimer (Right) in the wildtype and analog forms inside different lipid bilayers, with different insertion orientations,

and at different salt concentrations. Note, an is the abbreviation for analog, wt for wildtype,10PIP2 for POPC+10%PIP2 membrane, NaCl for salt concentration at 0.3 M, Z for initial orientation aligned on Z-axis, while head for alignment on head. PCPG is for POPC+POPG mixed membrane.

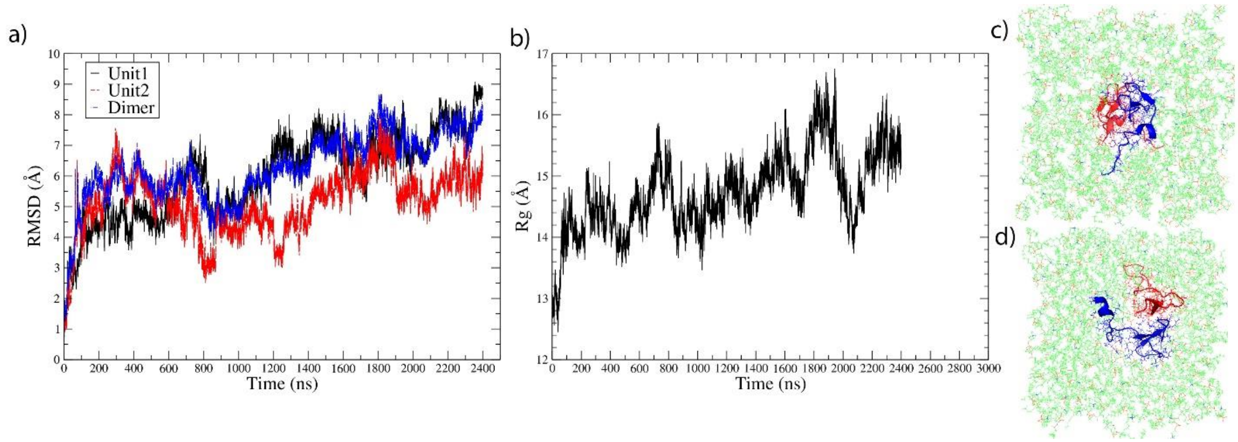

Figure S3. RMSD (a) and R_g (b) of the hBD-3 dimer in analog form and its units embedding inside the POPC+POPG mixed lipid bilayer, the initial and final structures of hBD-3 dimer in top-view is shown in c) and d).

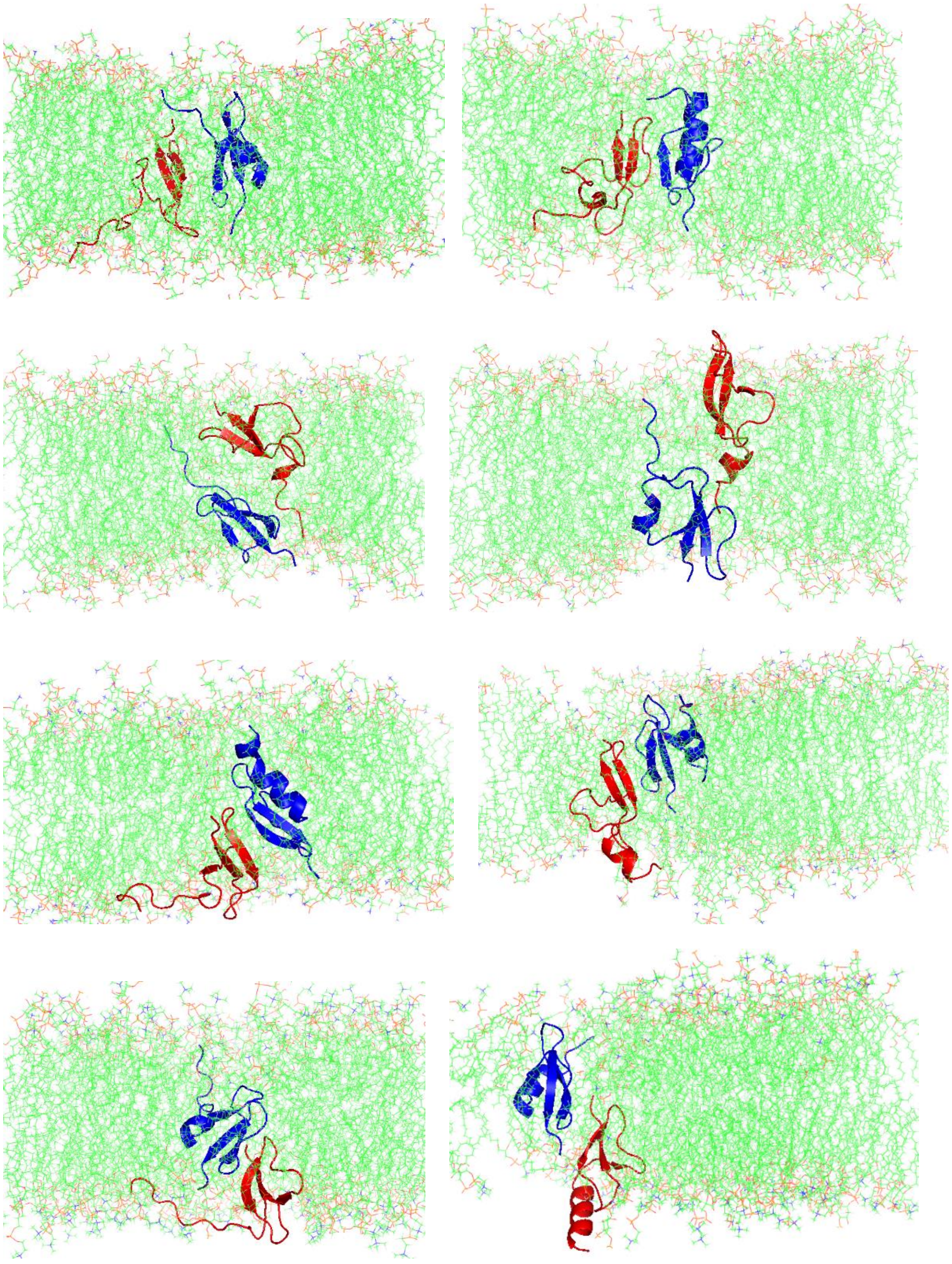

Figure S4. The last structures of the hBD-3 dimer in wildtype form embedded inside the Gram-positive bacterial lipid bilayer with the initial structure aligned in Z-axis (Top row) and in head (2nd row), in the Gram-negative bacterial lipid bilayer (3rd row) and in the POPC+10%PIP2 lipid bilayers (Bottom row) after 2400 or 1500 ns MD simulations. The systems at normal NaCl concentration are shown on the Left column, while those at the high NaCl concentration are shown on the Right column.

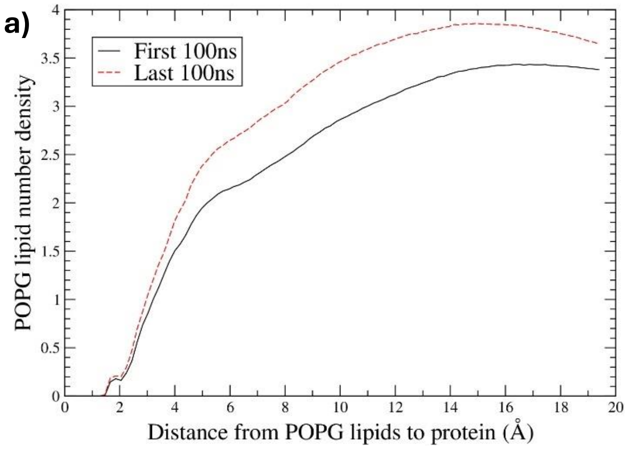

Figure S5. Comparison of the hBD3-POPG/PIP2 distance number density profiles in the POPC mixed with POPG lipid bilayer system (a) and POPC+10%PIP2 lipid bilayer system (b) based on the beginning 100 ns (in black solid line) and the last 100 ns (in red dashed line) of the 2.4 μs simulation.

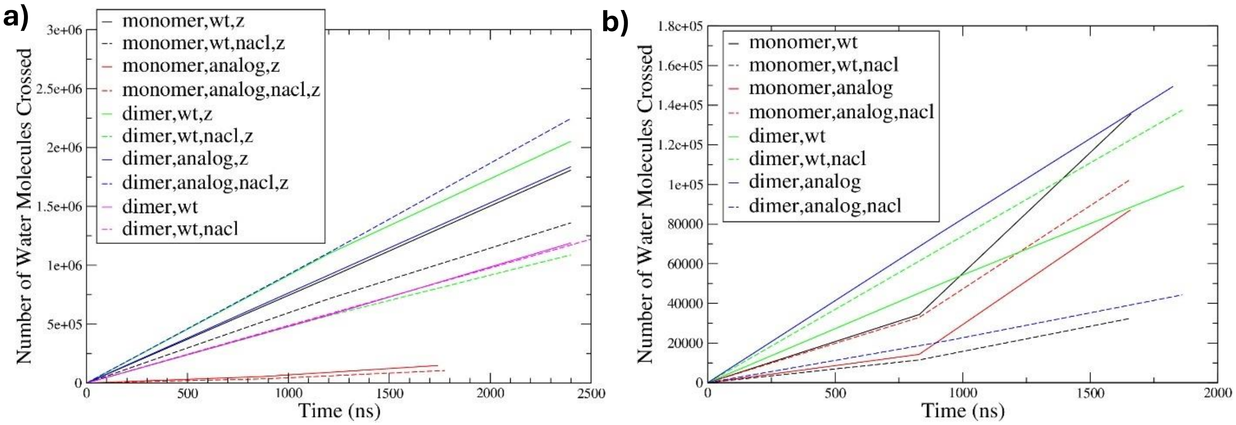

Figure S6. The number of water molecules crossed the G+ (a) and G- (b) membranes in hBD-3 monomer/dimer in both wildtype and analog systems during μs-long simulations. The system name description is consistent with the notes in the caption of Table I.

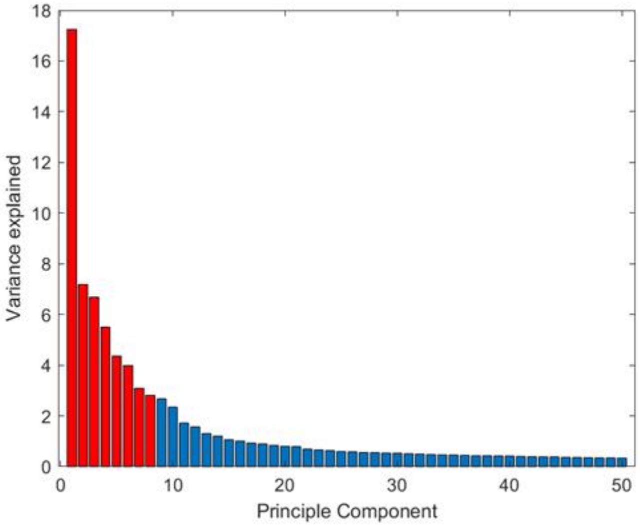

Figure S7. The variances (eigenvalues) of top 50 PCS. The variance explained for the first 8 PCs are shown in red, while the rest shown in blue. 50.8% of variance can be explained by the first 8 PCs.

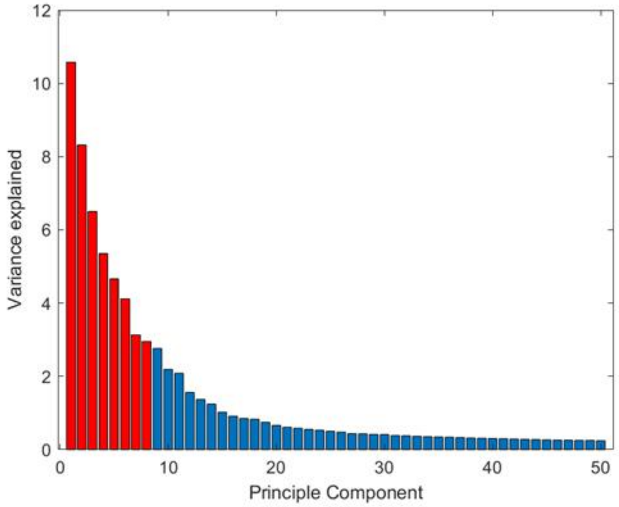

Figure S8. The variances of top 50 PCs. The variance explained for the first 8 PCs are shown in red, while the rest shown in blue. 45.6% of variance can be explained by the first 8 PCs.

3. Videos

VideoS1. During the 1.5 microsecond-long simulation, hBD-3 monomer in wildtype form (shown in red cartoon) moved from the center of the model Gram-negative bacterial lipid

bilayer to the surface (shown in cyan lines). The water molecules are not shown. The location and name of the file are:

figs-07-24-2025/moving-out of-membrane-process.mpg.
